# The Importance of Medical Students' Attitudes Regarding Cognitive Competence for Teaching Applied Statistics: Multi-Site Study and Meta-Analysis

**DOI:** 10.1371/journal.pone.0164439

**Published:** 2016-10-20

**Authors:** Natasa M. Milic, Srdjan Masic, Jelena Milin-Lazovic, Goran Trajkovic, Zoran Bukumiric, Marko Savic, Nikola V. Milic, Andja Cirkovic, Milan Gajic, Mirjana Kostic, Aleksandra Ilic, Dejana Stanisavljevic

**Affiliations:** 1 Institute for Medical Statistics and Informatics, Faculty of Medicine, University of Belgrade, Belgrade, Serbia; 2 Department of Internal Medicine, Division of Nephrology and Hypertension, Mayo Clinic, Rochester, United States of America; 3 Department of Primary Health Care and Public Health, Faculty of Medicine, University of East Sarajevo, Foca, Bosnia and Herzegovina; 4 Department of Public Health, Medical Faculty, University of Pristina, Kosovska Mitrovica, Serbia; University of Westminster, UNITED KINGDOM

## Abstract

**Background:**

The scientific community increasingly is recognizing the need to bolster standards of data analysis given the widespread concern that basic mistakes in data analysis are contributing to the irreproducibility of many published research findings. The aim of this study was to investigate students’ attitudes towards statistics within a multi-site medical educational context, monitor their changes and impact on student achievement. In addition, we performed a systematic review to better support our future pedagogical decisions in teaching applied statistics to medical students.

**Methods:**

A validated Serbian Survey of Attitudes Towards Statistics (SATS-36) questionnaire was administered to medical students attending obligatory introductory courses in biostatistics from three medical universities in the Western Balkans. A systematic review of peer-reviewed publications was performed through searches of Scopus, Web of Science, Science Direct, Medline, and APA databases through 1994. A meta-analysis was performed for the correlation coefficients between SATS component scores and statistics achievement. Pooled estimates were calculated using random effects models.

**Results:**

SATS-36 was completed by 461 medical students. Most of the students held positive attitudes towards statistics. Ability in mathematics and grade point average were associated in a multivariate regression model with the Cognitive Competence score, after adjusting for age, gender and computer ability. The results of 90 paired data showed that Affect, Cognitive Competence, and Effort scores demonstrated significant positive changes. The Cognitive Competence score showed the largest increase (M = 0.48, SD = 0.95). The positive correlation found between the Cognitive Competence score and students’ achievement (r = 0.41; p<0.001), was also shown in the meta-analysis (r = 0.37; 95% CI 0.32–0.41).

**Conclusion:**

Students' subjective attitudes regarding Cognitive Competence at the beginning of the biostatistics course, which were directly linked to mathematical knowledge, affected their attitudes at the end of the course that, in turn, influenced students' performance. This indicates the importance of positively changing not only students’ cognitive competency, but also their perceptions of gained competency during the biostatistics course.

## Background

The scientific community increasingly is recognizing the need to bolster standards of data analysis given the widespread concern that basic mistakes in data analysis are contributing to the irreproducibility of many published research findings, leading to a low number of preclinical studies that have been converted into clinical success [1]. Numerous studies demonstrating that insufficiently described methodology, lack of knowledge about statistical methods and their misuse are common in scientific publications, have led for calls to improve statistical training in biomedical education [2].

There are complex inter-relationships among various cognitive and non-cognitive factors that impact learning this subject. There is a growing demand to better understand these relationships in different educational environments [3]. What has been concluded previously is that a student’s background in mathematics is the main cognitive factor affecting his/her statistics achievement. Recent research has shown that non-cognitive factors such as students’ attitudes towards statistics also contribute to the understanding of statistical concepts and methods [4–8]. Evidence is mixed, in some papers relationships between achieved results and students' attitudes have been proven, but there are disagreements in the results of measuring attitudes regarding different competencies. Chiesi reported a correlation between cognitive and non-cognitive factors that affect achievement in statistics; students with less competence in mathematics had less confidence, displayed more negative feelings, and considered statistics to be more difficult than those students with better mathematics competence [5].

We implemented a blended learning model for medical students for teaching applied statistics with the aim of improving medical statistics education [9]. Our results provided empirical evidence to support educator decisions to implement different learning environments for teaching medical statistics. In line with this, we tested the psychometric properties of the Serbian version of the Survey of Attitudes Towards Statistics (SATS-36) in order to acquire a valid instrument to measure students’ attitudes inside the Serbian educational context [4]. As confirmatory factor analysis indicated that the SATS-36 demonstrated good indices for both reliability and validity, the questionnaire was used for the purpose of this analysis.

The aim of this study was to measure students’ attitudes towards statistics inside a multi-site medical educational context, monitor student attitude changes after an introductory course in medical statistics, and to explore the associations between cognitive and non-cognitive factors and statistics achievement. We also performed a systematic review of the literature for students' attitudes towards statistics, and a meta-analysis to evaluate the correlation between students' attitudes and statistics achievement to better support our future pedagogical decisions in teaching applied statistics to medical students.

## Methods

### Original study design and protocol

This was a prospective multicenter trial conducted with third year medical undergraduate students attending the Faculties of Medicine of the University of Belgrade, University of East Sarajevo and University of Pristina, who were enrolled in the obligatory introductory medical statistics course during the 2014–15 school year. The content of the course, learning objectives and course materials are described elsewhere [4]. The SATS-36 questionnaire [3] was used to measure students' attitudes towards statistics. The SATS contains 36 items (SATS-36) that are sorted into six attitude components: “Affect,” “Cognitive Competence,” “Value,” “Difficulty,” “Interest,” and “Effort.” Feedback for each item is graded from 1 (strongly disagree), through 4 (neutral), to 7 (strongly agree), using the 7 point Likert method. Both pre- and post-course versions of the SATS-36 were used (S1 File). Additional data included demographic and educational background information. Self-grading of abilities in mathematics and computers was also measured using the 7 point Likert response scale. High scores indicated that students had confidence in their overall mathematical or computer capabilities. The grade point average (GPA) (the mean value of grades achieved on previous exams ranged from a minimum of 6 to a maximum of 10) was also recorded for each student. The pre-course version of the SATS was administered to the participants in the classroom during the first statistics class for students attending on-site statistics classes, and for students attending a blended statistics course, the survey was conducted online. The response rate was 81%. Due to missing data, 10% of the questionnaires were excluded. The post-course version of the SATS was administered to 90 randomly selected students who attend the Faculty of Medicine, University of Belgrade for whom statistics achievement was also measured. Detailed methodology about students' evaluations in statistics has been described previously [4]. Briefly, the statistics achievement was reflected in the final statistics score (ranging from 0 to 100) that was identical for both learning modalities and consisted of: course activities throughout the year, a written knowledge test, and a final exam. Participation in this study was voluntary. Ethical approval for the study was obtained from the Institutional Review Boards (IRBs) of the Faculties of Medicine of Universities of Belgrade, Sarajevo and Pristina. Informed consent was obtained from all participants before conducting the study. For students attending on-site classes, verbal informed consent was obtained, which was recorded by a research assistant. Written consent was obtained from students attending on-line classes. As there was no potential harm to participants, the IRBs approved this consent procedure.

### Systematic review and meta-analysis

We performed a systematic review of peer-reviewed publications identified through searches of Scopus, Web of Science, Science Direct, Medline and APA databases from January 2015 to April 2016. Two biostatisticians with expertise in conducting systematic reviews and meta-analyses developed the search strategy containing the key words, “attitudes toward statistics” or “statistics attitudes” without restriction on methodology (S1 Table). There were no restrictions on publication language. Reference lists of papers that were included in the analysis were searched manually, as well as relevant reviews and editorials. Experts in the field were asked to provide information on potentially eligible studies. Two reviewers independently evaluated the eligibilities of all identified titles and abstracts. Studies were included in the full text screening if either reviewer identified the study as being potentially eligible, or if the abstract and title did not include sufficient information. Studies were eligible for full text screening if they had used any form of the SATS questionnaire to measure attitudes towards statistics and if they had been performed on a student population. The same reviewers independently performed full text screening to select articles for inclusion. Disagreements were resolved by consensus. Two reviewers independently abstracted the following data: 1) study design, 2) student population, 3) version of SATS questionnaire, 4) score for each SATS domain and 5) correlation coefficients for SATS domains and the statistics achievement. Reviewers used standardized forms and protocols when selecting and abstracting data. All independently extracted data were verified for congruence, and disagreements were resolved by consensus. Meta-analyses was performed for the correlation coefficients between 4 scale component scores (Cognitive Competence, Affect, Value and Difficulty) and statistics achievement. Individual study assessment for risk of bias was done based on outcome level (statistics achievement), according to RoBANS (Risk-of-bias assessment tool for nonrandomized studies) [10]. The systematic review was performed in accordance with the Preferred Reporting Items for Systematic Reviews (S2 File) [11].

### Statistical analysis

Descriptive statistics were calculated for baseline students’ characteristics, attitudes towards statistics components and final achievement scores. Cronbach’s alpha coefficient was used to assess the reliability of the Serbian version of SATS-36. Multivariable linear regression analysis was used to determine factors related to students’ attitudes regarding statistics. Differences between mean components scores at the beginning and at the end of the statistics course were analyzed using Students t-test. Pearson correlation coefficients were calculated to explore the relationships between the attitude component scores and statistics achievement. All tests were two-tailed. P<0.05 was considered statistically significant. The IBM SPSS 21 (Chicago, IL, 2012) package was used for these analyses.

The effect sizes for the meta-analysis were obtained by z-transformation of correlation coefficients extracted from studies identified through systematic review of the literature. Pooled estimates of correlation coefficients were calculated using random effects models [12,13]. The resulting pooled z-transformed correlation coefficients were back transformed (z to r transformation) to the level of the original coefficients for easier interpretation of results. Egger's test [14] and funnel plots were used to test for possible publication bias. τ^2^ and I^2^ were used to assess the heterogeneity of the included studies. All analyses were performed using R language for statistical computing [15] and R meta packages [16].

## Results

### Original study

The pretest SATS-36 was completed by 461 medical students: 336 from the Faculty of Medicine, University of Belgrade; 56 from the Faculty of Medicine, University of Sarajevo, Foca, and 69 from the Medical Faculty, University of Pristina, Kosovska Mitrovica. Mean age of the students was 21.24 ± 0.94 years, and most participants were female (58%). Participants reported good ability in mathematics (5.30 ± 1.51) and computers (4.89 ± 1.42). The current GPA was 8.47 ± 0.90 and the average statistics achievement score was 87.64 ± 8.89. Analysis of the internal consistency of the Serbian version of SATS-36 showed that the Cronbach’s alpha for the scale (items 1–36) was 0.87 and 0.77, for the pre-test and post-test SATS-36, respectively, indicating good scale reliability.

Most of the medical students held positive attitudes towards statistics. The average Affect component score was above neutral (4.6 ± 1.1) implying positive feelings. The highest mean scores were obtained for the Effort component (5.0 ± 0.7), Cognitive Competence (4.9 ± 1.0), and Interest (4.9 ± 1.3). According to the mean Value component score (4.8 ± 1.0), most students had positive perceptions about the value of statistics. The mean value of the Difficulty component scores indicated that students had neutral perceptions about the difficulty of statistics. Descriptive statistics of students’ attitudes regarding statistics for the SATS pre-test and post-test are presented in Table 1.

**Table 1:** Descriptive statistics of student attitudes towards statistics.

Author	Study design	Affect	Cognitive	Value	Difficulty	Interest	Effort
***Current study results*:**							
Milic N (2015)	pre test	4.6±1.1	4.9±1.0	4.8±1.0	4.0±0.7	4.9±1.3	5.0±0.7
	post test	4.8±1.2	5.4±1.2	4.8±1.1	3.9±0.9	4.7±1.3	5.5±1.1
***Systematic review results*:**							
Stanisavljevic D (2014)[4]	post test	4.2±1.1	4.9±1.2	4.1±1.1	3.6±0.8	4.0±1.5	4.9±1.2
Hannigan A (2014)***[17]	pre test	3.7(1.3)	4.6(1.3)	5.1(0.9)	3.4(0.9)	4.7(1.3)	6.0(0.9)
Zimprich D (2012)[26]	not clear	21.5±7.4	26.6±6.6	41.8±9.3	22.6±6.1	NA	NA
Zhang Y (2012)[6]	pre test	4.5±1.0	4.8±0.9	5.5±0.8	2.9±0.8	NA	NA
Schau C (2012)[18]	pre test	4.2±1.1	4.9±1.0	5.0±1.0	3.8±0.8	4.5 ±1.3	6.3±0.9
	post test	4.3±1.3	5.0±1.2	4.7±1.1	3.9±1.0	4.0±1.4	5.8±1.1
Hood M (2012)**[35]	post test	3.7	4.5	5.0	3.1	NA	NA
		(3.5,3.9)	(4.3,4.7)	(4.8,5.1)	(2.9,3.2)		
Harpe S E (2012)[19]	pre test	4.2±1.2	5.1±1.1	4.8±1.0	3.7±0.9	NA^#^	NA^#^
	post test	4.2±1.1	5.1±1.1	4.7±1.0	3.8±0.9		
Bond M E (2012)[20]	pre test	4.1±1.1	4.6±1.0	5.3±1.0	3.5±0.7	5.0±1.2	6.4±1.1
	post test	4.0±1.4	4.6±1.2	4.8±1.2	3.4±0.9	3.7±1.6	6.0±1.1
Carlson K A (2011)[21]	pre test	4.1±1.3	5.1±1.3	5.3±0.9	3.5±1.0	4.9±1.0	6.5±0.9
	post test	4.9±1.3	5.8±1.1	5.3±0.8	4.0±1.0	4.6±1.1	6.0±1.0
DeVaney TA (2010)[27]	pre test	3.6±1.3	4.6±1.2	4.9 ±0.9	3.2 ±0.9	NA	NA
	post test	4.1±1.5	4.9±1.2	4.9 ±1.1	3.4 ±0.9		
Coetzee S (2010)[22]	post test	4.6±1.3	5.3±1.2	4.9±1.1	3.4±1.1	NA^#^	NA^#^
Chiesi F(2010)[5]	pre test	22.3±4.7	26.2±5.2	44.6±6.7	23.2±4.0	NA	NA
	post test	24.0±5.2	30.2±4.6	45.8±6.4	25.1±4.0		
Wiberg M (2009)*[28]	post test	3.7±1.1	4.4±0.9	4.5±0.8	3.4±0.6	NA	NA
Dempster M (2009)[29]	pre test	20.8±6.5	26.4±6.5	46.5±8.0	23.7±6.0	NA	NA
	post test	22.0±7.7	25.5±6.3	42.0±9.4	23.1±5.8		
Mahmud Z (2008)*[30]	post test	4.8±1.0	4.9±1.0	5.2±0.9	3.5±0.9	NA	NA
Froelich AG (2008)[23]	pre test	4.5±1.0	5.3±0.8	5.2±0.9	4.0±0.6	4.5±1.1	6.1±0.8
	post test	4.6±1.2	5.3±1.0	4.9±1.0	4.1±0.9	4.2±1.4	5.8±1.0
Carnell LJ (2008)*[24]	pre test	4.4±1.1	5.2±0.8	5.0±0.8	3.8±0.4	4.6±1.0	5.8±1.0
	post test	4.3±1.5	5.1±1.4	4.6±1.2	3.9±1.0	3.6±1.5	5.2±1.5
Tempelaar DT(2007)[25]	pre test	4.5±1.1	5.1±0.9	5.1±0.8	3.6±0.8	5.1±1.0	6.4±0.7
Cashin SE (2005)[31]	pre test	24.9±8.7	30.0±6.7	47.5±8.8	23.7±6.2	NA	NA
	post test	26.7±8.5	30.6±6.9	47.2±8.7	24.6±6.9		
Nasser F (2004)[32]	not clear	4.8±1.3	5.3±1.1	4.7±1.1	3.4±1.1	NA	NA
Finney SJ (2003)[33]	pre test	24.5±6.9	29.7±6.5	43.7±8.2	27.0±5.2	NA	NA
	post test	26.5±7.9	31.8±7.2	41.0±9.7	28.4±7.1		
Faghihi F(1995)[34]	pre test	3.9±1.3	4.8±1.2	4.7±0.8	3.3±1.0	NA	NA
	post test	4.2±1.3	5.1±1.1	4.8±0.9	3.3±1.0		

NA- not applicable; NA^#^- not available

Data are presented as mean and sd

*pooled mean and sd

**mean (95%CI)

*** median (IQR)

In a multivariate regression model, self-rating of ability in mathematics and GPA were significantly associated with the Cognitive Competence score, after adjusting for age, gender and self-rating of ability in computers. Students with a better self-rating of ability in mathematics and higher current GPA had higher Cognitive Competence scores than those with poor self-ratings of ability in mathematics and current GPA. Self-rating of ability in computers was significantly associated with the Affect and Interest components, after adjusting for age, gender, and current GPA. Students with better self-ratings of ability in computers had higher Affect and Interest component scores than those with poor self-ratings of ability in computers. Age was significantly associated with the Effort component; older students had a higher Effort SATS-36 score compared to younger students. Gender was not significantly associated with the SATS-36 component scores (Table 2).

**Table 2:** Regression models of variables associated with student attitudes towards statistics (n = 461).

Variables	Affect		Cognitive		Value		Interest		Effort	
	Beta	p	Beta	p	Beta	p	Beta	p	Beta	p
Sex	0.121	0.065	0.033	0.614	0.073	0.270	0.085	0.204	0.107	0.113
Age	-0.126	0.064	-0.015	0.819	0.117	0.092	0.137	0.051	0.206	**0.003**
Mathematics	0.087	0.218	0.185	**0.009**	0.081	0.256	0.057	0.426	0.065	0.371
Computer skills	0.291	**<0.001**	0.095	0.152	0.084	0.214	0.178	**0.010**	0.100	0.144
GPA	0.016	0.822	0.229	**0.001**	0.260	**<0.001**	0.145	**0.049**	0.138	0.062

The results of 90 paired data showed that the Affect, Cognitive Competence and Effort scores demonstrated significant positive changes, while the changes in the Value and Interest scores were not significant (Table 3). The Cognitive Competence score showed the largest change (M = 0.48, SD = 0.95), indicating that students developed more positive attitudes towards their cognitive competence after completion of the statistics course. The mean Effort score increased by 0.44 (SD = 1.21), while the Affect score increased by 0.26 (SD = 1.08). Non-significant, very minor changes were obtained for the Value and Interest scores.

**Table 3:** Changes in student attitudes towards statistics after course completion (n = 90).

	Mean difference	SD (paired)	t-value	p-value
Affect	0.26	1.08	2.291	**0.024**
Cognitive	0.48	0.95	4.821	**< 0.001**
Value	-0.08	1.01	0.72	0.472
Difficulty	0.05	0.88	0.574	0.568
Interest	-0.23	1.15	1.924	0.058
Effort	0.44	1.21	3.441	**0.001**

The largest effect size was identified for the relationship between the Cognitive Competence component (r = 0.414) and statistics achievement. Students with more positive attitudes regarding cognitive competence towards statistics tended to perform better, as measured by the final statistics score. The Affect (r = 0.221) and Difficulty (r = 0.344) components were also related to students' achievement in statistics. The Value, Interest and Effort components had no significant correlations with statistics achievement (Table 4).

**Table 4:** Correlations between the attitude components scores and statistics achievement (n = 90).

Variable	r	p
Affect	0.221	**0.036**
Cognitive	0.414	**<0.001**
Value	0.115	0.282
Difficulty	0.344	**0.001**
Interest	-0.044	0.678
Effort	0.021	0.841

### Systematic review and meta-analysis

**Fig 1:**
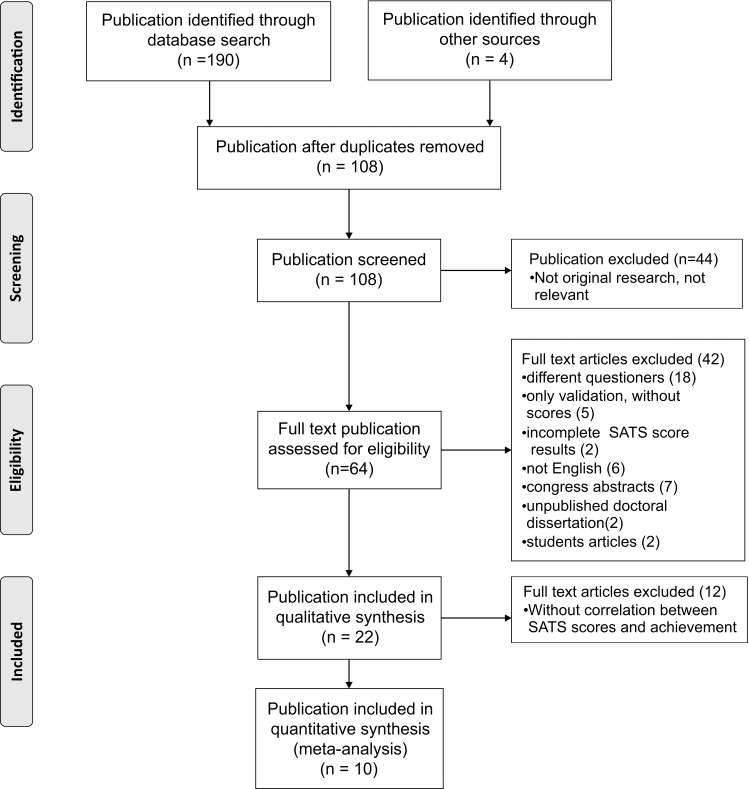
Flow of information through the different phases of a systematic review.

The total number of identified articles discussing student attitudes towards statistics was 108 after removing duplicates. Flow of information through the different phases of our systematic review is presented in Fig 1. Sixty-four full-text papers were assessed for eligibility, and 22 articles met the inclusion criteria and were included in the qualitative synthesis. The SATS-36 version was used in 10 (45.5%) publications [4,17–25], while 12 publications (54.5%) used the SATS-28 questionnaire [5,6,26–35]. Most studies (n = 16, 72.7%) assessed attitudes in undergraduate students [4,5,18–21,23–29,32,33,35]; graduate students were assessed in 3 studies (13.6%) [6,17,30], and 3 studies (13.6%) examined attitudes in both [22,31,34]. In 12 (54.5%) studies, both pre- and post- SATS were used to assess students' attitudes towards statistics [5,18–21,23,24,27,29,31,33,34]. Three studies (13.6%) used only the pre-test SATS version [6,17,25], 5 (22.7%) used the post-test [4,22,28,30,35], and in two studies (9.1%), it was not clear which SATS version was used [26,32]. For both pre- and post- test scores, summary measures for all scale components were extracted and presented in Table 1. The minimum value for the Affect component of SATS was 3.5 [29], and maximum was 4.8 [32]. The Cognitive component score had a lowest value of 4.4 [26,28,29] and a highest value of 5.8 [21]. The lowest Value score was found in undergraduate students 4.1 [4], while the highest score was 5.5, and identified in the graduate medical student population [6]. The range of students' attitudes for Difficulty was 2.9 [6] to 4.1 [23]. The minimum value for the Interest component was 3.6 [24] and the maximum was 4.9 [21]. The highest value for Effort was 6.5 [21] and the lowest score was 4.9 [4].

**Fig 2:**
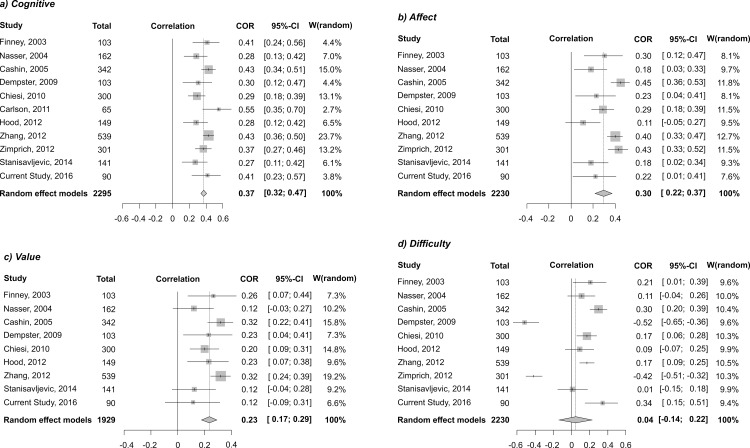
Forest plots for correlation coefficients (back transformed, z to r) between SATS components and statistics achievement a) Cognitive, b) Affect, c) Value and d) Difficulty component.

Ten studies from the systematic review [4–6,21,26,29,31–33,35] that reported correlation coefficients were included in the quantitative analysis. Results from this study were also included in the meta-analysis. Pooled estimates of correlation coefficients in random effects models were all positive and significant, except for the Difficulty component scores (Fig 2). The medium effect size correlation was found between Cognitive Competence and achievement (0.37; 95% CI 0.32–0.41; p<0.001), while small effect size correlations were found for Affect and achievement (0.30; 95% CI 0.22–0.37; p< 0.001), and Value and achievement (0.23; 95% CI 0.17–0.29; p<0.001). Individual study assessment for risk of bias is presented in S2 Table. Egger’s test suggested no publication bias (p>0.05) for all correlation coefficients. Funnel plots illustrated a symmetric distribution of z-transformed correlation coefficients, also indicating no evidence of publication bias. Examination of τ^2^ and I^2^ suggested low levels of heterogeneity for the Cognitive component and Value (τ^2^ = 0.003, I^2^ = 37.4%; and τ^2^ = 0.003, I^2^ = 38.4%, respectively), but high levels of heterogeneity for Affect and Difficulty (τ^2^ = 0.013, I^2^ = 72.7%; and τ^2^ = 0.079, I^2^ = 94.4%, respectively).

## Discussion

With the aim to investigate medical students’ attitudes towards statistics, and explore their impact on students' achievement, a validated Serbian SATS-36 questionnaire was administered to medical students attending an introductory course in biostatistics from three medical universities in the Western Balkans. A positive correlation between the Cognitive Competence component of students’ attitudes towards statistics and students’ achievement found in our original study, and also revealed in our meta-analyses, indicate the importance of measuring and evaluating medical students' attitudes about their cognitive competence in statistics.

Overall, our research has demonstrated that most medical students have positive attitudes regarding statistics. In line with previous studies, mean scores for the Affect, Effort, Cognitive Competence, Value and Interest components were all above neutral [4,6,18,23,24]. Unlike some other studies where students indicated more negative feelings [17,27,35], our original study results showed neutral student perceptions about the difficulty of statistics.

Consistent with results of previous studies regarding the association between mathematics background (cognitive factor) and attitudinal components (non-cognitive factors) [17,36,37], we found that self-rating of ability in mathematics and current GPA were related to the Cognitive Competence component. We also found that students' self-rating of ability in computers was significantly associated with the Affect and Interest components, while age was associated with the Effort component. In a previous study which enrolled medical postgraduate students, student computer skills were related to attitudes; students with better computer skills had more confidence in their ability to conduct complicated statistical computations [6]. As in the Hanningan et al. study [17], gender was not significantly associated with the SATS-36 component scores.

We have also demonstrated that students' attitudes towards statistics changed in a positive manner over the course; Affect, Cognitive and Effort scores showed significant positive changes, while the Value and Interest scores did not change significantly. The Cognitive Competence score showed the largest increase. These results are in contrast to the results of the Zhang et al. study, where the Affect and Cognitive Competence scores showed significant, albeit negative changes. Twelve studies, including ours, examined SATS scores longitudinally, i.e., before and after the statistics course. The scores for Cognitive Competence were found to be increased in several studies [5,21,27,33], but unchanged in others [19,20,29]. Increases in the Affect scores were seen in several studies [18,21,27], but not changed in others [19]. In our study, the scores for Value and Difficulty did not change, which is in agreement with other studies [19,27].

The relationships between the aforementioned cognitive and non-cognitive factors with statistics achievement may be quite important. Chiesi and Primi [5] reported that both a background in mathematics and attitudes towards statistics were related to statistics achievement. This was confirmed in our study by the positive correlations between mathematics knowledge, Cognitive Competence, Affect and Difficulty components, and statistics achievement. Among the non-cognitive factors, Cognitive Competence had the strongest correlation with achievement; implying that students who felt more competent in statistics demonstrated better statistics achievement. This finding is keeping with the literature reporting that Cognitive Competence was most strongly correlated with statistics achievement [5–7].

Separate meta-analyses were performed for 4 scale components in order to further explore all available evidence in the peer-reviewed literature for the correlations between scale component scores and statistics achievement. In keeping with the original study results, a moderate statistically significant positive relationship was found between the Cognitive Competence component and statistics achievement, demonstrating the importance of medical students' attitudes regarding their cognitive competence in statistics for their future knowledge achievement in statistics. The results of the performed meta-analyses also showed a significant, but small effect size relationship between the Affect and Value components and achievement, while there was no significant relationship between the Difficulty component and statistics achievement. Important pedagogical issues for teaching applied statistics can be derived from these findings. Both cognitive and non-cognitive factors should be covered when planning interventions to improve student performance. As these factors are interrelated, in general, and focused on attitudes implying cognitive competence, in particular, our strategies as educators should be aimed at boosting students' mathematical competence as well as their attitudes regarding the discipline.

## Conclusions

Mathematical knowledge acquired during previous education and attitudes regarding statistics had an effect on statistics achievement. Attitudes implying cognitive competence at the beginning of the biostatistics course, which are linked to mathematical knowledge, affected attitudes at the end of the course that, in turn, influenced student performance. This indicates the importance of positively changing not only student cognitive competency, but also student perception of gained cognitive competency during the course in biostatistics.

## Supporting Information

S1 File Pre- and post- course versions of Serbian SATS-36 questionnaire. (DOCX)

S2 File PRISMA checklist. (DOC)

S1 Table Search Strategy for Scopus Database. (DOCX)

S2 Table Individual study assessment for risk of bias according to RoBANS (Risk-of-bias assessment tool for nonrandomized studies). (DOCX)
