# Supplementary material for: The Importance of Medical Students' Attitudes Regarding Cognitive Competence for Teaching Applied Statistics: Multi-Site Study and Meta-Analysis
Source: PLoS One. 2016 Oct 20;11(10):e0164439. doi: 10.1371/journal.pone.0164439 (PMC5072734; doi:10.1371/journal.pone.0164439)
Supplement: S1 File — (DOCX) [file pone.0164439.s001.docx]

**UPITNIK NAMENJEN IDENTIFIKACIJI STAVOVA PREMA STATISTICI (SATS-36 pre-test)**

UPUTSTVO: Upitnik je namenjen identifikaciji vaših stavova o statistici. Za svaku stavku ponuđeno je 7 mogućih nivoa slaganja, od nivoa 1 (jako neslaganje) preko 4 (neutralan stav) do 7 (jako slaganje). Ako nemate određeni stav, izaberite nivo 4. Molimo Vas da pročitate svaku stavku. Obeležite jedan od ponuđenih nivoa koji najbolje odgovara Vašem slaganju ili neslaganju sa određenom stavkom. Pokušajte da ne razmišljate previše detaljno pri izboru, zaokružite odgovarajući nivo slaganja i pređite na sledeću stavku. Molimo Vas da odgovorite na sve stavke.

|  | Jako neslaganje | | Neutralan  stav | | | Jako slaganje | |
| --- | --- | --- | --- | --- | --- | --- | --- |
| Planiram da uradim seminar iz statistike. | 1 | 2 | 3 | 4 | 5 | 6 | 7 |
| Planiram da naporno učim tokom kursa iz statistike. | 1 | 2 | 3 | 4 | 5 | 6 | 7 |
| Dopašće mi se statistika. | 1 | 2 | 3 | 4 | 5 | 6 | 7 |
| Osećaću se nesigurno dok budem rešavao/la statističke probleme. | 1 | 2 | 3 | 4 | 5 | 6 | 7 |
| Imaću poteškoće u razumevanju statistike zbog svog načina razmišljanja. | 1 | 2 | 3 | 4 | 5 | 6 | 7 |
| Statističke formule su lake za razumevanje. | 1 | 2 | 3 | 4 | 5 | 6 | 7 |
| Statistika je bezvredna. | 1 | 2 | 3 | 4 | 5 | 6 | 7 |
| Statistika je komplikovana oblast. | 1 | 2 | 3 | 4 | 5 | 6 | 7 |
| Statistika treba da bude obavezni deo mog profesionalnog usavršavanja. | 1 | 2 | 3 | 4 | 5 | 6 | 7 |
| Statističke veštine će me učiniti korisnijim. | 1 | 2 | 3 | 4 | 5 | 6 | 7 |
| Ja nemam predstavu o tome šta će se dešavati tokom ovog kursa iz statistike. | 1 | 2 | 3 | 4 | 5 | 6 | 7 |
| Zainteresovan sam da se osposobim u razmenjivanju statističkih informacija. | 1 | 2 | 3 | 4 | 5 | 6 | 7 |
| Statistika nije korisna za tipičnog lekara. | 1 | 2 | 3 | 4 | 5 | 6 | 7 |
| Planiram da puno učim za test iz statistike. | 1 | 2 | 3 | 4 | 5 | 6 | 7 |
| Biću frustriran zbog testa iz statistike. | 1 | 2 | 3 | 4 | 5 | 6 | 7 |
| Statistički način mišljenja nije primenljiv u mom životu mimo moje profesije. | 1 | 2 | 3 | 4 | 5 | 6 | 7 |
| Koristim statistiku u svakodnevnom životu. | 1 | 2 | 3 | 4 | 5 | 6 | 7 |
| Biću pod stresom tokom trajanja ove nastave. | 1 | 2 | 3 | 4 | 5 | 6 | 7 |
| Uživaću tokom pohađanja kursa iz statistike. | 1 | 2 | 3 | 4 | 5 | 6 | 7 |
| Zainteresovan sam za primenu statistike. | 1 | 2 | 3 | 4 | 5 | 6 | 7 |
| Statistički zaključci se retko koriste u svakodnevnom životu. | 1 | 2 | 3 | 4 | 5 | 6 | 7 |
| Statistika je oblast koju većina brzo nauči. | 1 | 2 | 3 | 4 | 5 | 6 | 7 |
| Zainteresovan/a sam da razumem statističke koncepte. | 1 | 2 | 3 | 4 | 5 | 6 | 7 |
| Učenje statistike zahteva puno discipline. | 1 | 2 | 3 | 4 | 5 | 6 | 7 |
| Neću primenjivati statistiku u svom profesionalnom radu. | 1 | 2 | 3 | 4 | 5 | 6 | 7 |
| Napraviću puno matematičkih grešaka. | 1 | 2 | 3 | 4 | 5 | 6 | 7 |
| Planiram da prisustvujem svim časovima iz statistike. | 1 | 2 | 3 | 4 | 5 | 6 | 7 |
| Imam strah od statistike. | 1 | 2 | 3 | 4 | 5 | 6 | 7 |
| Zainteresovan sam da naučim statistiku. | 1 | 2 | 3 | 4 | 5 | 6 | 7 |
| Statistika uključuje puno izračunavanja. | 1 | 2 | 3 | 4 | 5 | 6 | 7 |
| Mogu da naučim statistiku. | 1 | 2 | 3 | 4 | 5 | 6 | 7 |
| Razumeću statističke formule. | 1 | 2 | 3 | 4 | 5 | 6 | 7 |
| Statistika je irelevantna u mom životu. | 1 | 2 | 3 | 4 | 5 | 6 | 7 |
| Statistika je oblast tehničke prirode. | 1 | 2 | 3 | 4 | 5 | 6 | 7 |
| Biće mi teško da razumem statističke koncepte. | 1 | 2 | 3 | 4 | 5 | 6 | 7 |
| Većina osoba mora da nauči nov način razmišljanja da bi se bavila statistikom. | 1 | 2 | 3 | 4 | 5 | 6 | 7 |

Molimo Vas da obratite pažnju da se za sledeće stavke razlikuju značenja mogućih nivoa slaganja.

|  | Potpuno neuspešan | | |  | Veoma uspešan | | |
| --- | --- | --- | --- | --- | --- | --- | --- |
| Koliko ste u dosadašnjem školovanju bili uspešni iz matematike? | 1 | 2 | 3 | 4 | 5 | 6 | 7 |
|  | Veoma loše | | |  | Jako dobro | | |
| Kako biste ocenili Vaše znanje iz matematike? | 1 | 2 | 3 | 4 | 5 | 6 | 7 |

Veoma loše Jako dobro

| Kako biste ocenili Vaše veštine u korišćenju kompjutera? | 1 | 2 | 3 | 4 | 5 | 6 | 7 |
| --- | --- | --- | --- | --- | --- | --- | --- |
|  | Nimalo | | |  | Jako puno | | |
| U oblasti u kojoj radite ili se nadate da ćete raditi u kojoj meri ćete koristiti statistiku ? | 1 | 2 | 3 | 4 | 5 | 6 | 7 |
|  | Nimalo | | |  | Veoma | | |
| Koliko ste sigurni da ćete savladati početni kurs iz statistike? | 1 | 2 | 3 | 4 | 5 | 6 | 7 |
|  | Nimalo | | |  | Veoma | | |
| Da ste mogli da birate koliko je verovatno da bi odabrali bilo koji kurs iz statistike tokom ovog školovanja? | 1 | 2 | 3 | 4 | 5 | 6 | 7 |

Nimalo Veoma

| Upotreba računara mi olakšava svakodnevni život | 1 | 2 | 3 | 4 | 5 | 6 | 7 |
| --- | --- | --- | --- | --- | --- | --- | --- |

UPUTSTVO: Za sledeća pitanja upišite odgovor u obliku broja ili zaokružite odgovarajući odgovor.

Pol:

1. Muški
2. Ženski

Starost (god.) ________

Prosečna ocena na redovnim studijama (ako ne znate procenite, a ako nemate unesite 99) ________

Koju ocenu očekujete da dobijete iz statistike?

1. 6
2. 7
3. 8
4. 9
5. 10

Oblik pohađanja nastave iz medicinske statistike:

1. Klasičan
2. Moodle

HVALA!

**UPITNIK NAMENJEN IDENTIFIKACIJI STAVOVA PREMA STATISTICI (SATS-36 post-test)**

UPUTSTVO: Upitnik je namenjen identifikaciji vaših stavova o statistici. Za svaku stavku ponuđeno je 7 mogućih nivoa slaganja, od nivoa 1 (jako neslaganje) preko 4 (neutralan stav) do 7 (jako slaganje). Ako nemate određeni stav, izaberite nivo 4. Molimo Vas da pročitate svaku stavku. Obeležite jedan od ponuđenih nivoa koji najbolje odgovara Vašem slaganju ili neslaganju sa određenom stavkom. Pokušajte da ne razmišljate previše detaljno pri izboru, zaokružite odgovarajući nivo slaganja i pređite na sledeću stavku. Molimo Vas da odgovorite na sve stavke.

|  | Jako neslaganje | | Neutralan  stav | | | Jako slaganje | |
| --- | --- | --- | --- | --- | --- | --- | --- |
| Trudio/la sam se da uradim seminar iz statistike. | 1 | 2 | 3 | 4 | 5 | 6 | 7 |
| Naporno sam učio/la tokom kursa iz statistike. | 1 | 2 | 3 | 4 | 5 | 6 | 7 |
| Dopada mi se statistika. | 1 | 2 | 3 | 4 | 5 | 6 | 7 |
| Osećam se nesigurno dok rešavam statističke probleme. | 1 | 2 | 3 | 4 | 5 | 6 | 7 |
| Imam poteškoće u razumevanju statistike zbog svog načina razmišljanja. | 1 | 2 | 3 | 4 | 5 | 6 | 7 |
| Statističke formule su lake za razumevanje. | 1 | 2 | 3 | 4 | 5 | 6 | 7 |
| Statistika je bezvredna. | 1 | 2 | 3 | 4 | 5 | 6 | 7 |
| Statistika je komplikovana oblast. | 1 | 2 | 3 | 4 | 5 | 6 | 7 |
| Statistika treba da bude obavezni deo mog profesionalnog usavršavanja. | 1 | 2 | 3 | 4 | 5 | 6 | 7 |
| Statističke veštine će me učiniti korisnijim. | 1 | 2 | 3 | 4 | 5 | 6 | 7 |
| Ja nemam predstavu o tome šta se dešava tokom ovog kursa iz statistike. | 1 | 2 | 3 | 4 | 5 | 6 | 7 |
| Zainteresovan sam da se osposobim u razmenjivanju statističkih informacija. | 1 | 2 | 3 | 4 | 5 | 6 | 7 |
| Statistika nije korisna za tipičnog lekara. | 1 | 2 | 3 | 4 | 5 | 6 | 7 |
| Trudio/la sam se da puno učim za test iz statistike. | 1 | 2 | 3 | 4 | 5 | 6 | 7 |
| Bio sam frustriran/na zbog testa iz statistike. | 1 | 2 | 3 | 4 | 5 | 6 | 7 |
| Statistički način mišljenja nije primenljiv u mom životu mimo moje profesije. | 1 | 2 | 3 | 4 | 5 | 6 | 7 |
| Koristim statistiku u svakodnevnom životu. | 1 | 2 | 3 | 4 | 5 | 6 | 7 |
| Ja sam pod stresom tokom trajanja ove nastave. | 1 | 2 | 3 | 4 | 5 | 6 | 7 |
| Ja uživam tokom pohađanja kursa iz statistike. | 1 | 2 | 3 | 4 | 5 | 6 | 7 |
| Zainteresovan/na sam za primenu statistike. | 1 | 2 | 3 | 4 | 5 | 6 | 7 |
| Statistički zaključci se retko koriste u svakodnevnom životu. | 1 | 2 | 3 | 4 | 5 | 6 | 7 |
| Statistika je oblast koju većina brzo nauči. | 1 | 2 | 3 | 4 | 5 | 6 | 7 |
| Zainteresovan/na sam da razumem statističke koncepte. | 1 | 2 | 3 | 4 | 5 | 6 | 7 |
| Učenje statistike zahteva puno discipline. | 1 | 2 | 3 | 4 | 5 | 6 | 7 |
| Neću primenjivati statistiku u svom profesionalnom radu. | 1 | 2 | 3 | 4 | 5 | 6 | 7 |
| Pravim puno matematičkih grešaka. | 1 | 2 | 3 | 4 | 5 | 6 | 7 |
| Trudio/la sam se da prisustvujem svim časovima iz statistike. | 1 | 2 | 3 | 4 | 5 | 6 | 7 |
| Imam strah od statistike. | 1 | 2 | 3 | 4 | 5 | 6 | 7 |
| Zainteresovan/na sam da naučim statistiku. | 1 | 2 | 3 | 4 | 5 | 6 | 7 |
| Statistika uključuje puno izračunavanja. | 1 | 2 | 3 | 4 | 5 | 6 | 7 |
| Mogu da naučim statistiku. | 1 | 2 | 3 | 4 | 5 | 6 | 7 |
| Razumem statističke formule. | 1 | 2 | 3 | 4 | 5 | 6 | 7 |
| Statistika je irelevantna u mom životu. | 1 | 2 | 3 | 4 | 5 | 6 | 7 |
| Statistika je oblast tehničke prirode. | 1 | 2 | 3 | 4 | 5 | 6 | 7 |
| Teško mi je da razumem statističke koncepte. | 1 | 2 | 3 | 4 | 5 | 6 | 7 |
| Većina osoba mora da nauči nov način razmišljanja da bi se bavila statistikom. | 1 | 2 | 3 | 4 | 5 | 6 | 7 |

Molimo Vas da obratite pažnju da se za sledeće stavke razlikuju značenja mogućih nivoa slaganja.

|  | Nimalo | | |  | Veoma | | |
| --- | --- | --- | --- | --- | --- | --- | --- |
| Koliko ste sigurni da ste savladali početni kurs iz statistike? | 1 | 2 | 3 | 4 | 5 | 6 | 7 |
|  | Nimalo | | |  | Veoma | | |
| Kada završite školovanje koliko ćete koristiti statistiku? | 1 | 2 | 3 | 4 | 5 | 6 | 7 |
|  | Nimalo | | |  | Veoma | | |
| Da možete da birate koliko je verovatno da biste odabrali sledeći kurs iz statistike? | 1 | 2 | 3 | 4 | 5 | 6 | 7 |
|  | Veoma  lako | | |  | Veoma  teško | | |
| Koliko Vam je teško gradivo ovog kursa iz statistike? | 1 | 2 | 3 | 4 | 5 | 6 | 7 |

UPUTSTVO: Za sledeća pitanja upišite odgovor u obliku broja ili zaokružite odgovarajući odgovor.

Broj poena na kolokvijumu iz statistike (0 do 20)________

Da li ste sigurni koju ocenu ćete dobiti iz statistike?

1. Da
2. Ne

Koju ocenu očekujete da dobijete iz statistike?

1. 6
2. 7
3. 8
4. 9
5. 10

U uobičajenoj nedelji, koliko ste sati mimo nastave utrošili na učenje statistike? ________

|  | Veoma  nizak | | |  | Veoma  visok | | |
| --- | --- | --- | --- | --- | --- | --- | --- |
| U prethodnom semestru, kako bi ste opisali ukupan nivo stresa? | 1 | 2 | 3 | 4 | 5 | 6 | 7 |

HVALA!
