# Supplementary material for: The Importance of Medical Students' Attitudes Regarding Cognitive Competence for Teaching Applied Statistics: Multi-Site Study and Meta-Analysis
Source: PLoS One. 2016 Oct 20;11(10):e0164439. doi: 10.1371/journal.pone.0164439 (PMC5072734; doi:10.1371/journal.pone.0164439)
Supplement: S1 Table — (DOCX) [file pone.0164439.s003.docx]

**S1 Table**. **Search Strategy for Scopus Database**

| **Scopus** |
| --- |
| **Search strategy** |
| TITLE-ABS-KEY ( **"attitude* toward* statistic*"**  OR  **"statistic* attitude*"** )   AND  PUBYEAR  >  **1994** |
