# Supplementary material for: The Importance of Medical Students' Attitudes Regarding Cognitive Competence for Teaching Applied Statistics: Multi-Site Study and Meta-Analysis
Source: PLoS One. 2016 Oct 20;11(10):e0164439. doi: 10.1371/journal.pone.0164439 (PMC5072734; doi:10.1371/journal.pone.0164439)
Supplement: S2 Table — (DOCX) [file pone.0164439.s004.docx]

**S2 Table. Individual study assessment for risk of bias according to RoBANS (Risk-of-bias assessment tool for nonrandomized studies)**

| **Study** | **Domains*** | | | | | |
| --- | --- | --- | --- | --- | --- | --- |
|  | **Selection of participants** | **Confounding variables** | **Measurement of exposure** | **Blinding of outcome assessments** | **Incomplete outcome data** | **Selective outcome reporting** |
| Finney SJ (2003) [33] | low | low | unclear | unclear | high | low |
| Nasser F (2004) [32] | low | high | low | unclear | low | low |
| Cashin SE (2005) [31] | low | low | low | unclear | unclear | low |
| Dempster M (2009) [29] | low | low | low | unclear | high | low |
| Chiesi F (2010) [5] | low | low | low | unclear | high | low |
| Carlson KA (2011) [21] | low | low | low | unclear | high | low |
| Hood M (2012) [35] | low | low | low | unclear | low | low |
| Zhang Y (2012) [6] | low | low | low | low | high | low |
| Zimprich D (2012) [26] | low | low | unclear | unclear | low | low |
| Stanisavljevic D (2014) [4] | low | high | low | low | low | low |

*Domain details

Selection of participants: Selection bias caused by the inadequate selection of participants.

Confounding variables: Selection bias caused by the inadequate confirmation and consideration of confounding variable.

Measurement of exposure: Performance bias caused by the inadequate measurement of exposure.

Blinding of outcome assessments: Detection bias caused by the inadequate blinding of outcome assessments.

Incomplete outcome data: Attrition bias caused by the inadequate handling of incomplete outcome data.

Selective outcome reporting: Reporting bias caused by the selective reporting of outcomes.
